# Automated Traffic Surveillance Using Existing Cameras on Transit Buses

**DOI:** 10.3390/s23115086

**Published:** 2023-05-26

**Authors:** Keith A. Redmill, Ekim Yurtsever, Rabi G. Mishalani, Benjamin Coifman, Mark R. McCord

**Affiliations:** 1 Department of Electrical and Computer Engineering, The Ohio State University, Columbus, OH 43210, USA; coifman.1@osu.edu; 2 Center for Automotive Research, The Ohio State University, Columbus, OH 43212, USA; yurtsever.2@osu.edu; 3 Department of Civil, Environmental, and Geodetic Engineering, The Ohio State University, Columbus, OH 43210, USA; mishalani.1@osu.edu (R.G.M.); mccord.2@osu.edu (M.R.M.)

**Keywords:** vehicle detection and tracking, traffic monitoring, computer vision, intelligent transportation systems

## Abstract

Millions of commuters face congestion as a part of their daily routines. Mitigating traffic congestion requires effective transportation planning, design, and management. Accurate traffic data are needed for informed decision making. As such, operating agencies deploy fixed-location and often temporary detectors on public roads to count passing vehicles. This traffic flow measurement is key to estimating demand throughout the network. However, fixed-location detectors are spatially sparse and do not cover the entirety of the road network, and temporary detectors are temporally sparse, providing often only a few days of measurements every few years. Against this backdrop, previous studies proposed that public transit bus fleets could be used as surveillance agents if additional sensors were installed, and the viability and accuracy of this methodology was established by manually processing video imagery recorded by cameras mounted on transit buses. In this paper, we propose to operationalize this traffic surveillance methodology for practical applications, leveraging the perception and localization sensors already deployed on these vehicles. We present an automatic, vision-based vehicle counting method applied to the video imagery recorded by cameras mounted on transit buses. First, a state-of-the-art 2D deep learning model detects objects frame by frame. Then, detected objects are tracked with the commonly used SORT method. The proposed counting logic converts tracking results to vehicle counts and real-world bird’s-eye-view trajectories. Using multiple hours of real-world video imagery obtained from in-service transit buses, we demonstrate that the proposed system can detect and track vehicles, distinguish parked vehicles from traffic participants, and count vehicles bidirectionally. Through an exhaustive ablation study and analysis under various weather conditions, it is shown that the proposed method can achieve high-accuracy vehicle counts.

## 1. Introduction

Ever-increasing demand and congestion on existing road networks necessitates effective transportation planning, design, and management. This informed decision making requires accurate traffic data. Most medium- to long-term traffic planning and modeling is focused on traffic demand, as quantified by traffic counts or flow. Traffic flow is generally measured by fixed-location counting, which can be accomplished using permanent detectors or, in many cases, temporary detectors or human observers. Many traditional traffic studies use temporary pneumatic tube or inductive loop technologies that are deployed for only a few days at each location, and each location is revisited only every three to five years. So, while traffic flow measurement is key to estimating demand throughout the network, the available data are spatially and temporally sparse and often out of date.

A large body of work has focused on automatic vision-based traffic surveillance with stationary cameras [1,2,3,4,5,6,7]. More recently, state-of-the-art vehicle detection performance has appreciably improved with Convolutional Neural Network (CNN)-based mature 2D object detectors such as Mask-RCNN [8], Yolo v3 [9], and Yolo v4 [10]. These models are feasible to deploy in real time. For most surveillance tasks, a processing rate of 4–5 frames per second is sufficient [11], which is achievable with these detectors. However, stationary, fixed-location cameras cannot cover the entirety of the road network.

Unmanned Aerial Vehicle (UAV) based automatic traffic surveillance [12,13,14,15] is a good alternative to stationary cameras. Drones can monitor a larger part of the network and track vehicles across multiple segments. However, UAV operation carries its own technical and regulatory limitations. Adverse climate affects flight dynamics substantially [15], and vehicle detection suffers from the lower resolution of camera views at higher flight elevations.

Recent studies proposed and developed the concept that public transit bus fleets could be used as surveillance agents [16,17,18]. Transit buses tend to operate on roadways with a greater concentration of businesses, residences, and people, and so they often cover many of the more heavily used streets that would be selected for a traditional traffic study. As discussed in the above noted studies, this method could also be deployed on other municipal service vehicles that might already be equipped with cameras; however, one advantage of using transit buses is that their large size allows cameras to be mounted higher above the ground, thus reducing the occurrence of occlusions.

Prior work on measuring traffic flow from moving vehicles has relied on manual counts or used dedicated probe vehicles equipped with lidar sensors [16,17]. Lidar sensors were used because the automatic processing of lidar data is readily achievable. However, unlike lidar, video sensors are already deployed on transit buses for liability, safety, and security purposes. The viability, validity, and accuracy of the use of video imagery recorded by cameras mounted on transit buses was established by manually counting vehicles captured in this imagery with the aid of a graphical user interface (GUI) [18]. However, for practical large-scale applications, the processing of video imagery and the counting of vehicles must be fully automated.

**Figure 1:**
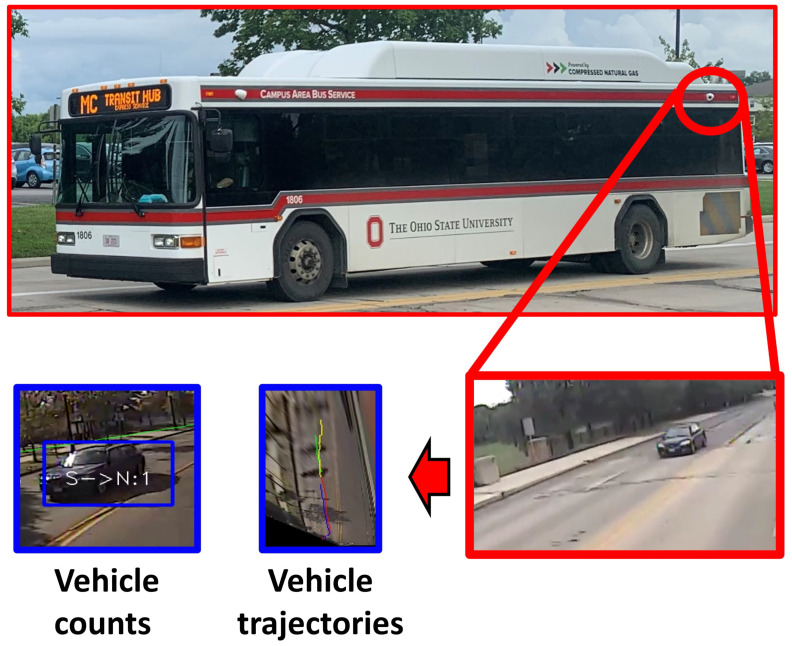
The proposed vehicle counting and trajectory extraction framework utilizes video cameras already mounted on existing transit buses for purposes other than traffic surveillance. Using transit buses as intelligent surveillance agents would thus be cost-effective and could increase traffic network coverage.

In this paper, we propose to operationalize this traffic surveillance methodology for practical applications, leveraging the perception and localization sensors already deployed on these vehicles. This paper develops a new, automatic, vision-based vehicle counting method applied to the video imagery recorded by cameras mounted on transit buses. To the best of our knowledge, this paper is the first effort to apply automated vision-based vehicle detection and counting techniques to video imagery recorded from cameras mounted on bus-transit vehicles. Figure 1 shows an example deployment of cameras on a transit bus, including example video imagery. This configuration is used in the experiments presented in this paper. By fully automating the vehicle counting process, this approach offers a potentially low-cost method to extend surveillance to considerably more of the traffic network than fixed-location sensors currently provide, while also providing more timely updates than temporary traffic study sensor deployments.

The main contributions of this study are the following:

- An automatic vision-based vehicle counting and trajectory extraction method using video imagery recorded by cameras mounted on transit buses.
- A real-world demonstration of the automated system and its validation using video from in-service buses. An extensive ablation study was conducted to determine the best components of the pipeline, including comparisons of state-of-the-art CNN object detectors, Kalman filtering, deep-learning-based trackers, and region-of-interest (ROI) strategies.

**Figure 2:**
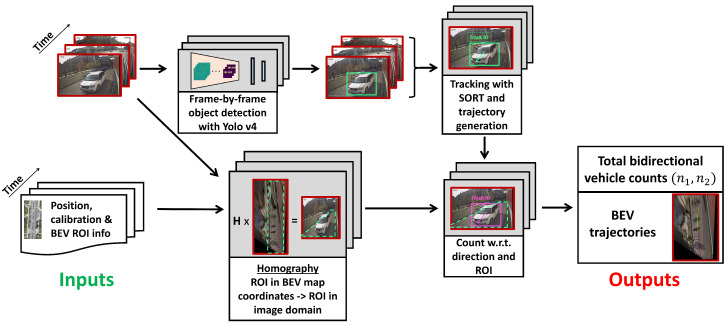
Overview of the proposed automated vehicle counting system. The objective is twofold: obtaining total bidirectional vehicle counts and extracting trajectories in bird’s-eye-view (BEV) coordinates. Our method uses streams of images, GNSS measurements, and predefined ROI information on a 2D map as inputs. The detection and tracking branch does not require geo-referencing, but it is necessary for counting and projecting trajectories in BEV world coordinates. The homography calibration needs to be performed only once. Only four point correspondences are required for the calibration process.

The main challenges in this endeavor are as follows: estimating vehicle trajectories from a limited observation window, robust vehicle detection and tracking while moving, localizing the sensor platform, and differentiating parked vehicles from traffic participants. Figure 2 shows an overview of the processing pipeline. First, images obtained from a monocular camera stream are processed with the automatic vehicle counter developed in this study. A Yolo v4 [10] deep CNN trained on the MS COCO dataset [19] detects vehicles, while SORT [20], a Kalman filter and Hungarian algorithm based tracker which solves an optimal assignment problem, generates unique tracking IDs for each detected vehicle. Next, using subsequent frames, the trajectory of each detected vehicle is projected onto the ground plane with a homographic perspective transformation. Then, each trajectory inside a predefined, geo-referenced region-of-interest (ROI) is counted with a direction indicator. The automatic vehicle counts are compared against human-annotated ground-truth counts for validation. In addition, an exhaustive ablation study is conducted to determine the best selection of 2D detectors, trackers, and ROI strategies.

## 2. Related work

### 2.1. Traffic Surveillance with Stationary Cameras

Automating vehicle detection and counting is crucial for increasing the efficiency of image-based surveillance technologies. A popular approach has been to decompose the problem into subproblems: detecting, tracking, and counting [2].

Detection: Conventional methods employ background–foreground difference to detect vehicles. Once a background frame, such as an empty road stretch, is recorded, the background can be subtracted from the query frames to detect vehicles on the road [3].

More recently, state-of-the-art deep-learning-based 2D object detectors such as Mask-RCNN [8], Yolo v3 [9], and Yolo v4 [10] have achieved remarkable scores on challenging object detection benchmarks [19,21]. Deep CNNs completely remove the background frame requirement. As such, pretrained, off-the-shelf deep CNNs can be deployed directly for vehicle detection and, if needed, vehicle classification tasks [6,7]. However, most vehicle detectors work on a single image frame, hence the need for tracking.

Tracking: Tracking is a critical step for associating detected objects across multiple frames and assigning a unique tracking ID to each vehicle. This step is essential for counting, as the same vehicle across multiple image frames should not be counted more than once. Once the vehicle is detected, a common approach is to track the bounding box with a Kalman filter [22].

SORT [20] is a more recent Kalman filter and Hungarian algorithm based tracker. Deep SORT [23] is an extension of SORT with a deep association metric, using a CNN-based feature extractor to associate objects with similar features across frames. Deep-SORT-based vehicle detectors [24] can achieve state-of-the-art performance.

Counting: The final component of the processing pipeline is counting. Usually, lane semantics with ROI reasoning [4] are used to define a counting area. This area is then used to decide which vehicles to count as traffic participants.

### 2.2. Traffic Surveillance with UAVs

Automated vehicle detection has progressed significantly over the years. However, stationary cameras cannot cover the entirety of the road network. The shortcomings of fixed-location, stationary cameras can be circumvented by using a UAV as a sensor platform [12,13,14,15]. Operating regulations have somewhat relaxed, and the cost of UAVs has fallen significantly over the past decade, allowing them to become a more cost-efficient solution for airborne surveillance.

Similar trends such as using Yolo [25] and Faster RCNN [26] are prevalent for UAV-based traffic surveillance. An important design criterion is flight altitude. There are three flight categories: low (up to 70 m) [27], mid-range (80–120 m) [28], and high (100–150 m) [29] altitude flight.

UAV-based surveillance systems suffer from lower resolutions and smaller observed vehicle sizes. In addition, since the viewing angle is top-down, vehicles’ distinguishing features cannot be observed. As such, off-the-shelf object detectors cannot be deployed directly. The deep learning models must be trained with a top-down object detection dataset [30].

UAV-based traffic surveillance is promising. However, adverse weather affects flight dynamics negatively [15], and safety concerns are a limiting factor for the usability of airborne drones.

### 2.3. Traffic Surveillance with Probe Ground Vehicles

The general concept of using moving vehicles to determine traffic variables is not new. It is common practice to use the floating car method to measure travel times and delays for arterial traffic studies [31,32] and to use probe vehicles to measure travel times in real time [33,34,35,36,37,38,39,40]. However, these approaches focus only on measuring the probe vehicle’s travel time.

This study focuses on the estimation of traffic flow rather than travel times. Traffic flow estimation requires counting other vehicles. Although the theoretical foundations of such a framework were laid decades ago [41], an operational fully automated system has not yet been realized and deployed in a practical large-scale setting. While there are recent efforts to extend cellphone tracking to estimate traffic volumes [42,43], these approaches are limited by the fact that there is not a one-to-one match between cellphones and vehicles.

### 2.4. Traffic Flow Using Moving Observers

The main idea behind the moving observer method is to estimate hourly traffic volume on a finite road section while the observer is traversing it. Recent studies have made progress in this regard. Specifically, one study used an instrumented vehicle equipped with lidar sensors to emulate a transit bus [16,17]. Lidar sensors and data were used because they are readily processed, with limited computing power, to automatically detect vehicles. The detected vehicles were then converted to counts, which in turn were transformed into estimates of traffic flows. However, modern transit buses are already equipped with video cameras for purposes other than traffic flow estimation, rather than with lidar sensors. Therefore, in a follow-on study [18], counts from video imagery recorded by cameras mounted on transit buses, which had been extracted manually using a GUI, were used to show and establish the viability and validity of estimating accurate traffic flows from the such imagery.

Another recent study focused on applying moving observer techniques to estimate traffic flow using automated vehicles and a data-driven approach [44]. However, a practical large-scale implementation of such a moving observer surveillance system is not possible without a method that can entirely automatically extract vehicle trajectories and count vehicles from a limited observation window.

We aim to fill this gap in the literature with the proposed automatic vehicle trajectory extraction and counting methodology. In principle, the methodology developed herein for counting vehicles from existing cameras on transit buses could be transferred to existing cameras on public service, maintenance, or safety vehicles and even private automobiles equipped with cameras already being used for driver assist, adaptive cruise control, or vehicle automation, e.g., [44].

## 3. Method

As mentioned previously, our work focuses solely on automatically obtaining vehicle counts and extracting trajectories using computer vision. What follows are detailed descriptions of the problem and the various steps of the developed methodology.

### 3.1. Problem Formulation

Given an observation at time *t* as $O_{t}=\left(I_{t},p_{t}^{\mathrm{bus}}\right)$, where $I\in Z^{H\times W\times 3}$ is an image captured by a monocular camera mounted on the bus and $p\in R^{2}$ is the position of the bus on a top-down 2D map, the first objective is to find $f_{1}:S\to \left(n_{1},n_{2}\right)$, a function that maps a sequence of observations $S=(O_{1},\cdots ,O_{N})$ to the total number of vehicles counted traveling in each direction $\left(n_{1},n_{2}\right)$. The second objective is to find $f_{2}:S\to {\left\{T_{i}\right\}}_{m}$, which provides the *m* tuples of detected vehicles’ trajectories. The trajectory of detected vehicle *i* is given with $T_{i}=\left\{\left(p_{i,j},t_{i,j}\right)|j\in \left(1,2\cdots ,J\right)\right\}$, where *J* is the total number of frames in which vehicle *i* was observed by the bus and $p_{i,j}$ is the bird’s-eye-view real-world position vector of vehicle *i*.

The overview of the solution is shown in Figure 2. The proposed algorithm, Detect-Track-Project-Count (DTPC), is given in Algorithm 1. The implementation details of the system are presented in Section 4.
**Algorithm 1:** Detect-Track-Project-Count: DTPC(S,H,R)
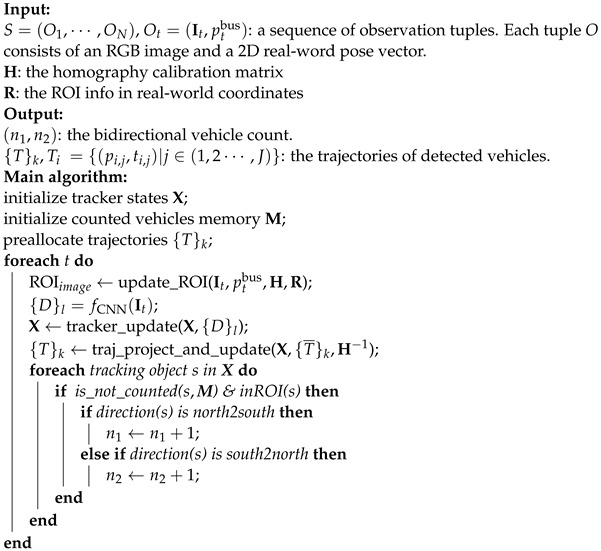


### 3.2. Two-Dimensional Detection

A vehicle detection *D* is defined as D=(b,l,c), where $b=(\left(u_{1},v_{1}\right),\left(u_{2},v_{2}\right),\left(u_{3},v_{3}\right),\left(u_{4},v_{4}\right))$ contains the bounding box corners in pixel coordinates, *l* is the class label (e.g., car, bus, or truck), and *c* is the confidence of the detection. A 2D CNN $f_{\mathrm{CNN}}:I\to {\left\{D\right\}}_{q}$ maps each image I captured by the bus camera to *q* tuples of detections *D*. This processing is performed on individual image frames. The object classification information is used to avoid counting pedestrians and bicycles but could also be used in other studies.

The networks commonly known as Mask-RCNN [8], Yolo v3 [9], and Yolo v4 [10], pretrained on the MS COCO dataset [19], are employed for object detection. Based on the ablation study presented subsequently, the best-performing detector was identified as Yolo v4.

### 3.3. Tracking

The goal of tracking is to associate independent frame-by-frame detection results across time. This step is essential for trajectory extraction, which is needed in order to avoid counting the same vehicle more than once.

SORT [20], a fast and reliable algorithm that uses Kalman filtering and the Hungarian algorithm to solve the assignment problem, is employed for the tracking task. First, define the state vector as

(1) $$\begin{array}{c} x={\left(u_{c},v_{c},s,r,\dot{u},\dot{v},\dot{s}\right)}^{⊺} \end{array}$$

where $u_{c}=u_{1}+\left(u_{3}-u_{1}\right)/2$ and $v_{c}=v_{2}+\left(v_{4}-v_{2}\right)/2$ are the bounding box center coordinates derived from the resultant 2D detection bounding boxes, $s=\left(u_{3}-u_{1}\right)\times \left(v_{4}-v_{2}\right)$ is its area, $r=\left(u_{3}-u_{1}\right)/\left(v_{4}-v_{2}\right)$ is its aspect ratio, and $\dot{u},\dot{v},$ and $\dot{s}$ are the corresponding first derivatives with respect to time.

The next step consists of associating each detection to already existing target boxes with unique tracking IDs. This subproblem can be formulated as an optimal assignment matching problem where the matching cost is the Intersection-over-Union (IoU) value between a detection box and a target box i.e.,

(2) $$\begin{array}{c} \mathrm{IoU}=\frac{Area\;of\;Intersection}{Area\;of\;Union}. \end{array}$$

This problem can be solved with the Hungarian algorithm [45].

After each detection is assigned to a target, the target state is updated with a Kalman filter [46]. The Kalman filter assumes the following dynamical model:

(3) $$\begin{array}{c} x_{k}=F_{k}x_{k-1}+B_{k}u_{k}+w_{k}. \end{array}$$

where, $F_{k}$ is the state transition matrix, $B_{k}$ is the control input matrix, $u_{k}$ is the control vector, and $w_{k}$ is normally distributed noise. The Kalman filter recursively estimates the current state from the previous state and the current actual observation as follows:

(4) $$\begin{array}{c} {\hat{x}}_{k|k-1}=F_{k}{\hat{x}}_{k-1|k-1}+B_{k}u_{k} \end{array}$$

(5) $$\begin{array}{c} P_{k|k-1}=F_{k}P_{k-1|k-1}F_{k}^{⊺}+Q_{k} \end{array}$$

where **P** is the predicted covariance matrix and **Q** is the covariance of the multivariate normal noise distribution. Details of the update step can be found in the original Kalman filter paper [46].

**Figure 3:**
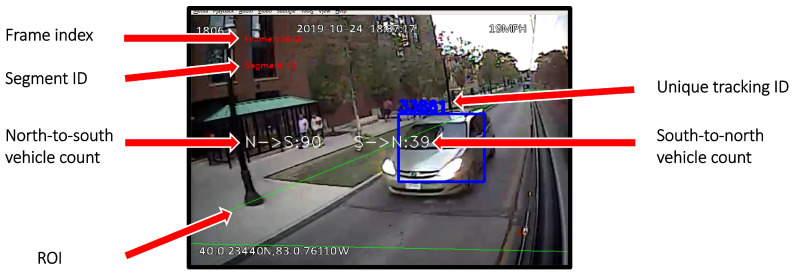
An example of a detected, tracked, and counted vehicle. The proposed system outputs bidirectional traffic counts. The segment ID indicates the current road segment occupied by the bus.

Deep SORT [23] was also considered as an alternative to SORT [20]; however, SORT gave better results. An example of a detected, tracked, and counted vehicle is shown in Figure 3.

### 3.4. Geo-Referencing and Homography

The proposed method utilizes geo-referencing and homographic calibration to count vehicles in the desired ROI and transforms the detected vehicles’ trajectories from pixel coordinates to real-world coordinates.

GNSS measurement data are used to localize the bus with a pose vector $p_{t}^{\mathrm{bus}}$ at time *t*. A predefined database contains geo-referenced map information about the road network and divides each road into road segments with a predefined geometry, including the lane widths for each segment. Road segments tend to run from one intersection to another but can also be divided at points where the road topology changes significantly, for example, when a lane divides. This information is used to build the ROI in BEV real-world coordinates for each road segment.

**Figure 4:**
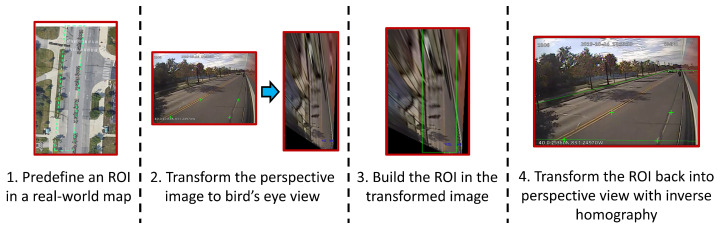
ROI alignment with homography. A predefined ROI in a 2D BEV world-map can be utilized for any camera angle with a planar homography transformation. The camera only needs to be calibrated once with four point correspondences.

The pixel coordinates of each detection can be transformed into real-world coordinates with planar homography using

(6) $$s\left[\begin{array}{c} u\\ v\\ 1 \end{array}\right]=H\left[\begin{array}{c} x\\ y\\ 1 \end{array}\right]$$

since planar homography is up-to-scale with a scaling factor. **H**, the homography matrix, has eight degrees of freedom. Hence, **H** can be determined from four real-world image point correspondences [47]. Finding four point correspondences is fairly straightforward for urban road scenes. Standard road markings and the width of a lane are used to estimate **H**. The homographic calibration process is shown in Figure 4.

Once **H** is obtained, the inverse projection can be easily achieved with the inverse homography matrix $H^{-1}$. Inverse homography is used to convert a BEV real-world ROI to a perspective ROI for the image plane. After obtaining the perspective ROI, vehicles on the corresponding regions can be counted.

### 3.5. Counting

The objective of counting is to count each unique tracked vehicle once if it is a traffic participant and not a parked vehicle, travels in the corresponding travel direction, and is within the ROI.

The 2D object detector’s output is considered only if the inferred class *l* = {car,bus,truck} and the bounding box center is within the ROI. Thus, at each counting time *t*, using $u_{c,i}$ and $v_{c,i}$, the bounding box center coordinates of each tracked vehicle *i*, the count in the top-to-bottom direction (i.e., opposite the direction of travel of the bus) is updated incrementally $n_{1}\leftarrow n_{1}+1$ if the one-step sequence $\left(u_{c,i,t-1},v_{c,i,t-1}\right),\left(u_{c,i,t},v_{c,i,t}\right)$ of the image domain trajectory of vehicle *i* satisfies $\mathrm{sgn}(u_{c,i,t}-u_{c,i,t-1})=1$ and vehicle *i* was not counted before. In a similar fashion, the count in the bottom-to-top direction (i.e., in the direction of travel of the bus) is updated incrementally $n_{2}\leftarrow n_{2}+1$ if $\mathrm{sgn}(u_{c,i,t}-u_{c,i,t-1})=-1$ and vehicle *i* was not counted before. The ROI alignment for counting purposes is shown in steps 3 and 4 of Figure 4.

**Figure 5:**
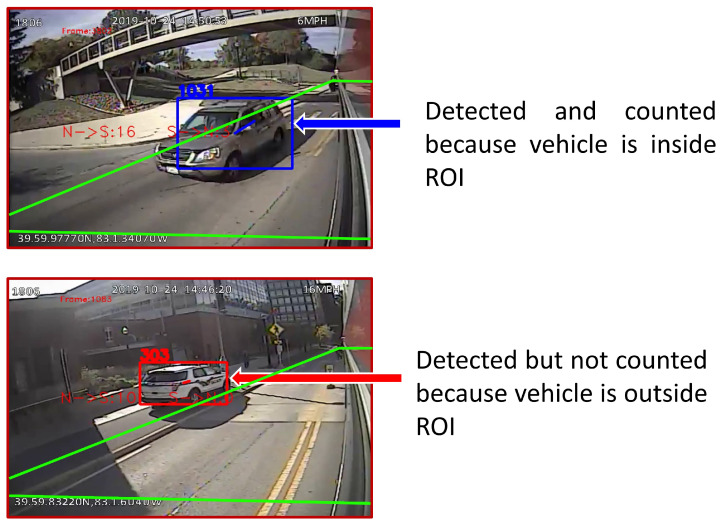
Distinguishing parked vehicles from traffic participants. Since the sensor platform is moving, excluding parked vehicles from the total count is not trivial. Using an ROI alleviates this issue.

Distinguishing parked vehicles from traffic flow participants is achieved by the ROI. This distinction and the difference between detecting and counting vehicles is illustrated in Figure 5.

### 3.6. Trajectory Extraction in BEV Real-World Coordinates

**Figure 6:**
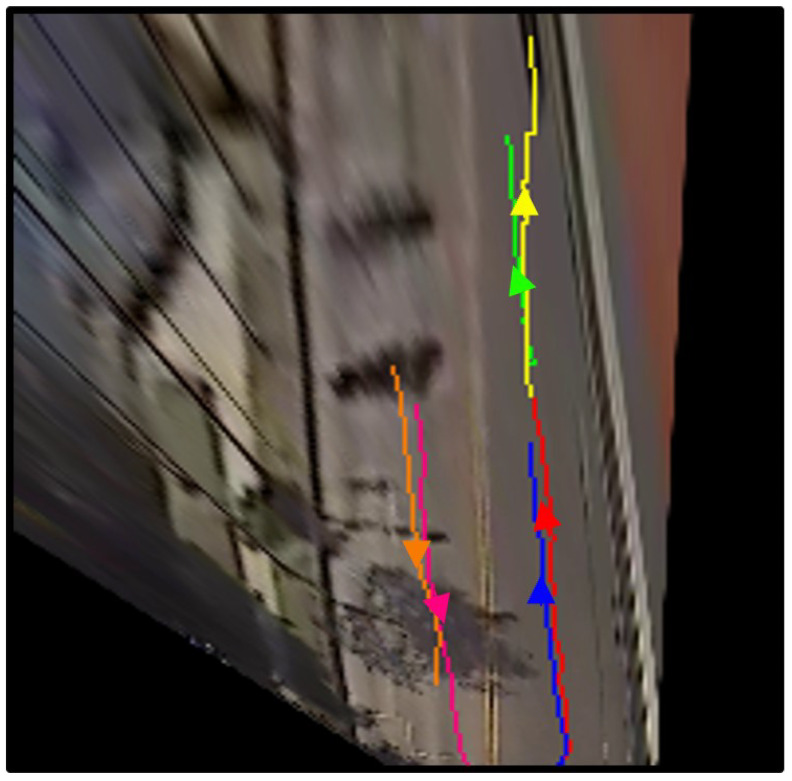
Extracted trajectories in real-world BEV coordinates for an observation window of approximately 5 s are shown. Six vehicles were detected, tracked, and have been projected onto a BEV plane. The trajectory of each vehicle is shown in a distinct color.

Finally, for each tracked unique vehicle, a trajectory in BEV real-world coordinates relative to the bus is built by transforming the image-domain coordinates using the inverse homography projection. The image domain trajectory of vehicle *i*, ${\bar{T}}_{i}=\left\{\left(u_{c,i,t},v_{c,i,t}\right)|t\in \left(t_{1},t_{2}\cdots ,t_{K}\right)\right\}$, is transformed into $T_{i}$, with inverse homography, ${\left(x_{c,i,t},y_{c,i,t},1\right)}^{⊺}=$$H^{-1}$${\left(u_{c,i,t},v_{c,i,t},1\right)}^{⊺}$. An example of extracted trajectories is illustrated in Figure 6.

Trajectory extraction in BEV real-world coordinates is not strictly necessary for the purpose of counting unique vehicles, which could be achieved without such transformation. However, it is quite valuable for understanding the continuous behavior of traffic participants, including their speed, and therefore could be used as input to other studies.

## 4. Experimental Evaluation

### 4.1. Transit Bus

The Ohio State University owns and operates the Campus Area Bus Service (CABS) system consisting of a fleet of about 50 40-foot transit buses that operate on and in the vicinity of the OSU campus, serving around five million passengers annually (pre-COVID-19). As is the case for many transit systems, the buses are equipped with an Automatic Vehicle Location (AVL) system that includes GNSS sensors for operational and real-time information purposes and several interior- and exterior-facing monocular cameras that were installed for liability, safety, and security purposes. The proposed method depends on having an external view angle wide enough to capture the motion pattern of the surrounding vehicles. Figure 3, Figure 4 and Figure 5 show sample image frames recorded by these cameras.

Video imagery collected from CABS buses while in service are used to implement and test the developed method. We chose to use the left side-view camera in this study because it is mounted higher than the front-view camera, which both reduces the potential for occlusions caused by other vehicles and improves the view of multiple traffic lanes to the side of the bus, particularly on wider multilane roads or divided roads with a median. Moreover, video from these cameras is captured at a lower resolution, which significantly reduces the size of the video files that needed to be offloaded, transferred, and stored. The video footage was recorded at 10 frames per second with a resolution of 696 × 478.

### 4.2. Implementation Details

The proposed algorithm was implemented in Python using OpenCV, Tensorflow, and Pytorch libraries. For 2D detectors, the original implementations of Mask-RCNN [8], Yolo v3 [9], and Yolo v4 [10] were used. All models were pretrained on the MS COCO dataset [19]. Experiments were conducted with an NVIDIA RTX 2080 GPU.

High-speed real-time applications are not within the scope of this study. For surface road traffic surveillance purposes, low frame rates are sufficient. The video files used in this study were downloaded from the buses after they complete their trips. However, it would be possible to perform the required calculations using edge computing installed on the bus or even integrated with the camera. This is a topic for future study.

### 4.3. Data Collection and Annotation

**Figure 7:**
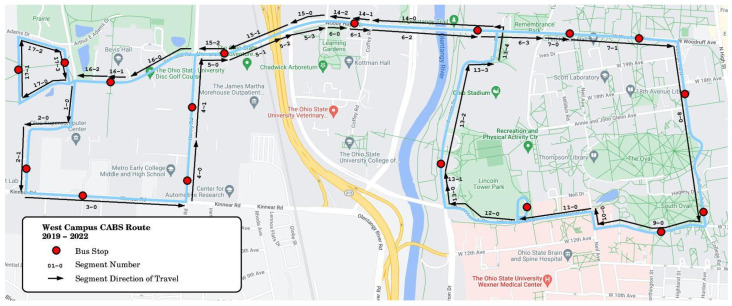
Data were collected in the main campus area of the Ohio State University in Columbus, OH. The route contains 4-lane and 2-lane public roads and on-campus streets. Some streets were divided by a median strip. The direction of travel arrows indicates the travel direction of the bus. Details of the route can be found in [18].

A total of 3.5 hours of video footage, collected during October 2019, was used for the experimental evaluation of the ablation study. An additional 3 hours of video footage, collected during March 2022, was used for the evaluation of the impacts of adverse weather. The West Campus CABS route on which the footage was recorded traverses four-lane and two-lane public roads. The route is shown in Figure 7. As described above, this route was divided into road segments which tend to run from one intersection to another but may be divided at other points, such as when the road topology changes significantly. This can include points at which the number of lanes change or, in the case of this route, areas in which a tree-lined median strip obscures the camera view. Using this map database, sections with a tree-lined median strip and occlusions were excluded from the study. For each road segment, an ROI was defined with respect to road topology and lane semantics to specify the counting area. As a result, a total of 55 bus pass and roadway segment combinations were used in the ablation study evaluation analysis.

We note that should the bus not follow its prescribed route or the route changes, the algorithm can detect and report this due to the map database, as well as remove or ignore those portions of traveled road that are off-route or for which the road geometry information needed to form an ROI is not available.

The video footage was processed by human annotators in order to extract ground-truth vehicle counts. Annotators used a GUI to incrementally increase the total vehicle count for each oncoming unique vehicle and to capture the corresponding video frame number. Annotators counted vehicles once and only if the vehicle passed a virtual horizontal line drawn on the screen of the GUI while traveling in the direction opposite to that of the bus.

After annotation, the video frames, counts, and GNSS coordinates were synchronized for comparison with the results of the developed image-processing-based fully automated method.

### 4.4. Evaluation Metrics

First, for each of the 55 bus pass and segment combination, ground-truth counts and inferred counts using the developed automatic counting method were compared with one another. In addition to examining a scatter plot that depicts this comparison, four metrics were considered, namely difference $c_{gt}-c_{i}$, absolute difference $|c_{gt}-c_{i}|$, absolute relative difference $|c_{gt}-c_{i}|/c_{gt}$, and relative difference $\left(c_{gt}-c_{i}\right)/c_{gt}$, where $c_{gt}$ is the ground-truth count, and $c_{i}$ is the inferred count.

The sample mean, sample median, and empirical Cumulative Distribution Function (eCDF) were calculated for the proposed and alternative automated baseline methods.

### 4.5. Ablation Study

An extensive ablation study was conducted to identify the best alternative among multiple methods that could be applied to each phase of the counting system. All the combinations of modules from the following list were compared with one another using the sample mean and sample median of the evaluation metrics to find the better performing combinations:

- 2D Detectors: Mask-RCNN [8], Yolo v3 [9], and Yolo v4. [10]
- Tracking option: SORT [20] and Deep SORT [23].
- ROI strategy: No ROI, Generic (single) ROI, and Dynamic Homography ROI.

The Generic ROI was defined based on a standard US two-lane road in perspective view. Each combination option is denoted by the sequence of uppercase first letters of the name of the modules. For example, Y4SGR stands for the Yolo 4 detector, SORT tracker, and Generic ROI combination. The proposed method is denoted with “Proposed” and stands for the Yolo 4 detector, SORT tracker, and Dynamic ROI combination.

## 5. Results and Discussion

### 5.1. Ablation study

**Figure 8:**
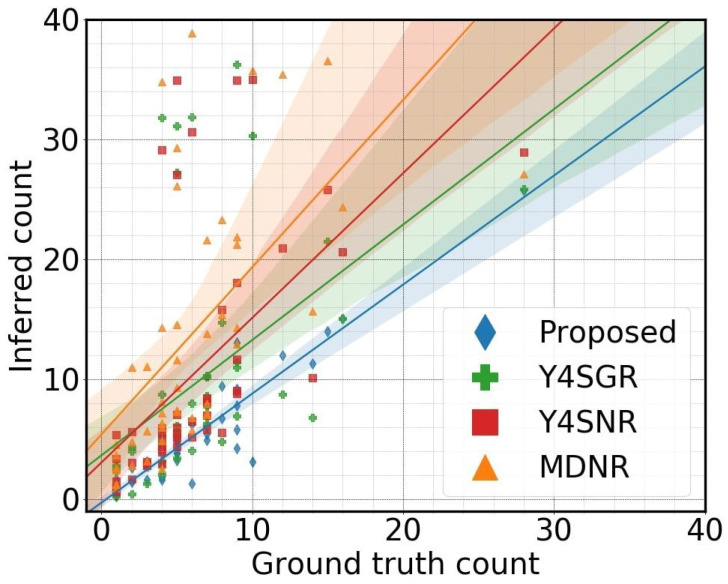
Inferred count versus ground-truth count: Y4SDR (Proposed) indicates Yolo 4 detector, SORT tracker, and Dynamic ROI; Y4SGR indicates Yolo 4 detector, SORT tracker, and Generic ROI; Y4SNR indicates Yolo 4 detector, SORT tracker, and No ROI; MDNR indicates Mask-RCNN detector, Deep SORT tracker, and No ROI. An ideal counter should be on the identity line (y=x). The proposed method is close to ideal, while other methods overcount.

Considering all combinations of tools, the proposed method, consisting of the Yolo v4 object detector, SORT tracker, and Dynamic Homography ROI, was found to be the best based on the sample mean, sample median, and eCDF of the four evaluation metrics. Figure 8 shows pairs of inferred counts plotted against ground-truth counts for each of the 55 road segment passes for select combinations of modules (including the best-performing one among all combinations) that reflect a wide range of performance. For a perfect automatic counting system, the scatter plot of inferred counts versus ground truth should be on the identity line (*y* = *x*). A regression line was estimated for each combination. The estimated lines are shown in Figure 8, along with their confidence limits. Clearly, the proposed Y4SDR outperforms the other four combinations shown in Figure 8 by a substantial margin.

As expected, the proposed homography-derived ROI performs better than the No ROI and the Generic ROI modules. The latter two ROI modules lead to over counts because of their limitations in distinguishing traffic participants from irrelevant vehicles. In contrast, the dynamic ROI allows for counting vehicles only in the pertinent parts of the road network, thus omitting irrelevant vehicles, which in this study consisted mostly of vehicles parked on the side of the roads or in adjacent parking lots. This result validates the developed bird’s-eye-view inverse projection approach to defining dynamic ROIs suitable to each roadway segment.

**Figure 9:**
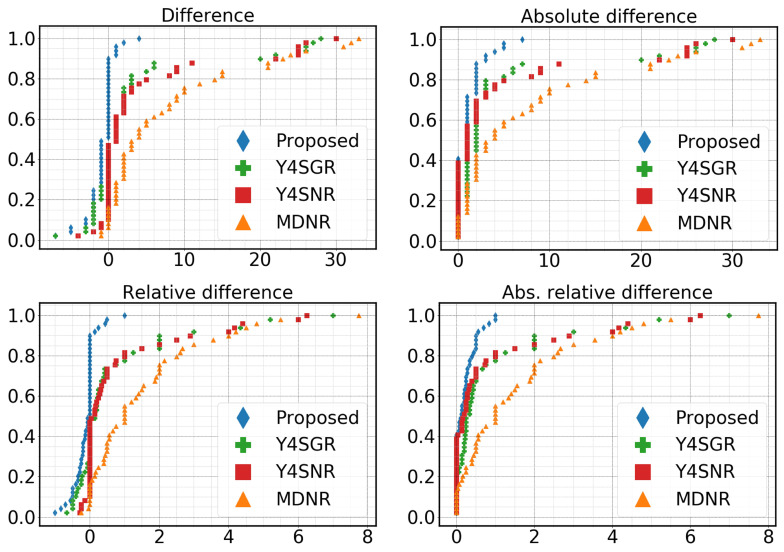
Empirical Cumulative Distribution Functions (eCDFs) of the proposed method and three other alternative combinations of modules. The proposed method consistently makes fewer errors (difference from the ground-truth count) across multiple road segments.

Figure 9 shows the eCDF plots of the four different functions from the four different trackers. In addition to confirming the overall superiority of the Y4SDR combination, these plots also indicate that this combination is highly reliable, as is evident from the thin tails of the distributions.

Summary results for all combinations of modules are shown in Table 1. Specifically, the sample mean and sample median of absolute differences and absolute relative differences are given for each considered detector, tracker, and ROI combination. Across all combinations of detector and ROI options, SORT outperformed Deep SORT consistently. This result indicates that the deep association metric used by Deep SORT needs more training to be efficient. In addition, across all combinations of tracker and ROI options, the detectors Yolo v3 and Yolo v4 performed similarly and better than Mask-RCNN. This result indicates that two-stage detectors, such as Mask-RCNN, are prone to overfitting to the training data more than single-stage detectors. Moreover, it can be seen that for all detector and tracker combinations, the proposed dynamic ROI option outperforms the other two ROI options by a large margin, as one might expect. Finally, from the results in Table 1, the Y4SDR combination is seen to perform the best among all combinations.

### 5.2. Impacts of Adverse Weather and Lighting

**Figure 10:**
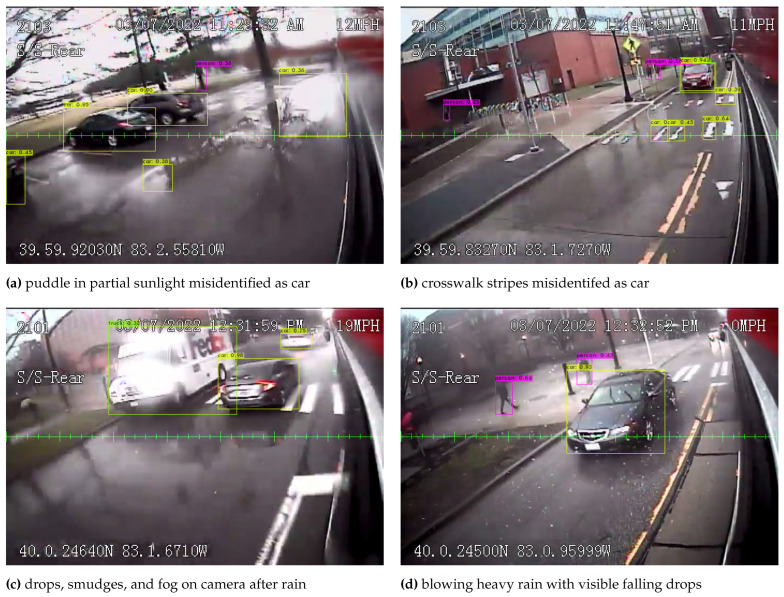
Examples of challenges for image processing results during and after heavy rain.

Having determined the best algorithm to implement from the results of the ablation study, one may also consider the performance of both the image processing and the overall counting system in the presence of inclement weather, including rain or post-rain conditions with puddles and irregular lighting effects. Inclement weather exposes several issues with an image-processing-based system, including darker and more varied lighting conditions, reflective puddles that can be misidentified as vehicles (Figure 10a), roadway markings misidentified as vehicles (Figure 10b), water droplets and smudges formed from dust or dirt and water partially obscuring some portions of the image (Figure 10c), and finally, blowing heavy rain, or possibly snow, with falling drops that can be seen in the images (Figure 10d).

We compared human-extracted ground truth with the automated extraction and counting results from videos of several loops of the West Campus route both on dry, cloudless, sunny days and days with rainfall varying from a light drizzle to heavy thunderstorms, which also included post-rain periods of time with breaking clouds that provided both darker and occasionally sunlit conditions. All the videos were acquired from actual in-service transit vehicles during the middle of the day.

Qualitatively, rainfall and wet conditions caused more transient—lasting only for one to two frames— inaccurate detection events to occur in the base image processing portion of the algorithm, including vehicles not detected, misclassified vehicles and other objects, and double detections when a vehicle is split into two overlapping objects, along with incorrectly identifying a greater number of background elements (e.g., puddles and road markings) as vehicles or objects. However, these transient events generally make little difference in the final vehicle counts, as the overall algorithm employs both filtering to eliminate unreasonable image processing results and tracking within the region of interest, such that a vehicle is declared present and counted only after a fairly complete track is established from the point it enters to the point it departs the camera’s field of view. This approach, in general terms, imposes continuity requirements, causing transient random events to be discarded unless they are so severe as to make it impossible to match and track a vehicle as it passes through the images, thereby improving the robustness of the approach.

More problematic, however, are persistent artifacts such as water droplets or smudges formed by wet dust and dirt, which often appear on the camera lens or enclosure cover after the rain stops and the lens begins to dry. For example, in one five-minute period of one loop, there was a large smudge on the left side at the vertical center of the lens, which obscured the region of the image where vehicles several lanes to the left of the transit bus tended to cross through and leave the image frame, causing them to not be counted due to incomplete tracking.

We note that these are general problems that can affect most video and image processing systems—if the lens is occluded you cannot see anything in that region of the image. It would be possible in future work to implement a method to dynamically detect when the lens is occluded and note the temporary exclusion of those regions from counts while the occlusion persists. As a final note, heavy rain was also observed to clean the lens.

Quantitatively, we present the results of the automated extraction and counting experiments in Table 2 for both the dry, clear, sunny loops and the rainy and post-rain loops. The table presents the percentage of correctly counted vehicles, the percentage of vehicles missed due to not being detected at all or detected too infrequently to build a sufficient track, the percentage of vehicles not detected due to being substantially occluded by another vehicle (this is not actually a weather-related event but is included for completeness), and the percentage of vehicles not counted due to a smudge or water droplet covering part of the camera lens. The final columns of Table 2 indicate the percentage of double-counted vehicles and the percentage of false detections or identifications that persisted long enough to be tracked and incorrectly counted. These are two impacts of transient image processing failures that are not always detected, at present, by our filtering and tracking algorithms.

As can be seen in Table 2, the overall effects of poor weather conditions result in only a minor increase in the errors committed by this system.

## 6. Conclusions

This paper introduced and evaluated a fully automatic vision-based method for counting and tracking vehicles captured in video imagery from cameras mounted on buses for the purpose of estimating traffic flows on roadway segments using a previously developed moving observer methodology. The proposed method was implemented and tested using imagery from in-service transit buses, and its feasibility and accuracy was shown through experimental validation. Ablation studies were conducted to identify the best selection of alternative modules for the automated method.

The proposed method can be directly integrated into existing and future ground-vehicle-based traffic surveillance approaches. Furthermore, since cameras are ubiquitous, the proposed method can be utilized for different applications.

Reimagining public transit buses as data collection platforms has great promise. With widespread deployment of the previously developed moving observer methodology facilitated by the full automation of vehicle counting proposed in this paper, a new dimension can be added to intelligent traffic surveillance. Combined with more conventional methods, such as fixed location and the emerging possibilities of UAV-based surveillance, spatial and temporal coverage of roadway networks can be increased and made more comprehensive. This three-pronged approach has the potential of achieving close to full-coverage traffic surveillance in the future.

Future work could focus on further comprehensive evaluation of the method presented here under more varied conditions, subsequent refinements, and the use of edge computing technologies to perform the image processing and automatic counting onboard the buses in real time. Another potential extension would involve coordinated tracking of vehicles across multiple buses, although this raises certain social and political privacy issues that would need to be addressed. Finally, there could be significant uses and value in vehicle motion and classification information, potential extensions to include tracking and counting bicycles, motorcycles, and pedestrians, and the eventual integration into smart city infrastructure deployments.

## Figures and Tables

**Table 1:** Comparison of alternative configurations of modules. The proposed method with a Yolo 4 detector, SORT tracker, and Dynamic ROI has the lowest difference (error) from the ground-truth counts. A good ROI ensures the exclusion of parked and irrelevant vehicles from the count.

		Absolute Error		Abs. Relative Error	
		**Tracker Option**		**Tracker Option**	
**ROI Option**	**Detector Option**	**SORT [20]**	**Deep SORT [23]**	**SORT [20]**	**Deep SORT [23]**
Dynamic ROI (Proposed)		μ (M)	μ (M)	μ (M)	μ (M)
	Yolo v3 [9]	1.24 (1)	2.02 (1)	0.22 (0.14)	0.32 (0.28)
	Yolo v4 [10]	1.18 (1)	1.67 (1)	0.21 (0.12)	(0.32, 0.25)
	Mask-RCNN [8]	2.34 (1)	2.32 (1)	0.39 (0.25)	0.46 (0.26)
Generic ROI					
	Yolo v3 [9]	4.16 (1)	4.48 (2)	0.75 (0.25)	0.84 (0.33)
	Yolo v4 [10]	4.30 (1)	4.59 (2)	0.75 (0.25)	0.89 (0.33)
	Mask-RCNN [8]	5.40 (2)	6.24 (4)	0.94 (0.5)	1.26 (0.86)
No ROI					
	Yolo v3 [9]	4.44 (1)	5.04 (1)	0.85 (0.25)	0.98 (0.28)
	Yolo v4 [10]	4.69 (1)	5.14 (2)	0.85 (0.2)	1.02 (0.35)
	Mask-RCNN [8]	6.02 (3)	7.89 (4)	1.05 (0.5)	1.53 (1)

μ: mean, M: median.

**Table 2:** Comparison of vehicle detection and counting results for clear/dry versus rain/post-rain weather conditions.

Weather	True Vehicle Counts	Missed Vehicles	Missed Vehicles Due to Occlusion by other Vehicles	Missed Vehicles Due to Smudge or Drops on Lens	Double-Counted Vehicles	Falsely Counted as Vehicle
	%	%	%	%	%	%
Dry/Clear/Sunny	90.7%	4.6%	4.6%	-	-	0.9%
Rain/Post-Rain	88.9%	5.9%	2.8%	2.3%	2.0%	1.7%

## Data Availability

The original video data used in this study as well as the manually extracted ground truth records are available in the Zenodo repository at https://zenodo.org/record/7955464.

## Abbreviations

The following abbreviations are used in this manuscript:

2D	Two-dimensional
BEV	Birds eye view
CABS	Campus Area Bus Service (OSU)
CNN	Convolutional neural network
eCDF	Empirical cumulative distribution function
GNSS	Global navigation satellite system
GUI	Graphical user interface
OSU	The Ohio State University
ROI	Region of interest
UAV	Unmanned aerial vehicle
YOLO	You Only Look Once

## References

[1] Coifman B., Beymer D., McLauchlan P., Malik J. (1998). A real-time computer vision system for vehicle tracking and traffic surveillance. Transp. Res. Part C Emerg. Technol..

[2] Alpatov B.A., Babayan P.V., Ershov M.D. Vehicle detection and counting system for real-time traffic surveillance. Proceedings of the 2018 7th Mediterranean Conference on Embedded Computing (MECO).

[3] Li D., Liang B., Zhang W. Real-time moving vehicle detection, tracking, and counting system implemented with OpenCV. Proceedings of the 2014 4th IEEE International Conference on Information Science and Technology.

[4] Wang K., Li Z., Yao Q., Huang W., Wang F.Y. An automated vehicle counting system for traffic surveillance. Proceedings of the 2007 IEEE International Conference on Vehicular Electronics and Safety.

[5] Felici-Castell S., García-Pineda M., Segura-Garcia J., Fayos-Jordan R., Lopez-Ballester J. (2021). Adaptive live video streaming on low-cost wireless multihop networks for road traffic surveillance in smart cities. Future Gener. Comput. Syst..

[6] Lin J.P., Sun M.T. A YOLO-based traffic counting system. Proceedings of the 2018 Conference on Technologies and Applications of Artificial Intelligence (TAAI).

[7] Zhu J., Li X., Jin P., Xu Q., Sun Z., Song X. (2021). MME-YOLO: Multi-sensor multi-level enhanced YOLO for robust vehicle detection in traffic surveillance. Sensors.

[8] He K., Gkioxari G., Dollár P., Girshick R. Mask R-CNN. Proceedings of the IEEE International Conference on Computer Vision.

[9] Redmon J., Farhadi A. (2018). Yolov3: An incremental improvement. arXiv.

[10] Bochkovskiy A., Wang C.Y., Liao H.Y.M. (2020). Yolov4: Optimal speed and accuracy of object detection. arXiv.

[11] Tian B., Morris B.T., Tang M., Liu Y., Yao Y., Gou C., Shen D., Tang S. (2014). Hierarchical and networked vehicle surveillance in ITS: A survey. IEEE Trans. Intell. Transp. Syst..

[12] Coifman B., McCord M., Mishalani R.G., Iswalt M., Ji Y. (2006). Roadway traffic monitoring from an unmanned aerial vehicle. IEE Proc. Intell. Transp. Syst..

[13] Zhang Y., Zhu D., Wang P., Zhang G., Leung H. (2020). Vision-based vehicle detection for VideoSAR surveillance using low-rank plus sparse three-term decomposition. IEEE Trans. Veh. Technol..

[14] Bozcan I., Kayacan E. Au-air: A multi-modal unmanned aerial vehicle dataset for low altitude traffic surveillance. Proceedings of the 2020 IEEE International Conference on Robotics and Automation (ICRA).

[15] Srivastava S., Narayan S., Mittal S. (2021). A survey of deep learning techniques for vehicle detection from UAV images. J. Syst. Archit..

[16] Redmill K.A., Coifman B., McCord M., Mishalani R.G. Using transit or municipal vehicles as moving observer platforms for large scale collection of traffic and transportation system information. Proceedings of the 2011 14th International IEEE Conference on Intelligent Transportation Systems (ITSC).

[17] Coifman B., Redmill K., Yang R., Mishalani R., McCord M. (2017). Municipal Vehicles as Sensor Platforms to Monitor Roadway Traffic. Transp. Res. Rec..

[18] McCord M.R., Mishalani R.G., Coifman B. (2020). Using Municipal Vehicles as Sensor Platforms to Monitor the Health and Performance of the Traffic Control System. Final Research Report, Mobility 21 A USDOT National University Transportation Center.

[19] Lin T.Y., Maire M., Belongie S., Hays J., Perona P., Ramanan D., Dollár P., Zitnick C.L. (2014). Microsoft COCO: Common objects in context. Proceedings of the European Conference on Computer Vision.

[20] Bewley A., Ge Z., Ott L., Ramos F., Upcroft B. Simple online and realtime tracking. Proceedings of the 2016 IEEE International Conference on Image Processing (ICIP).

[21] Krizhevsky A., Sutskever I., Hinton G.E. (2012). Imagenet classification with deep convolutional neural networks. Adv. Neural Inf. Process. Syst..

[22] Indrabayu, Bakti R.Y., Areni I.S., Prayogi A.A. Vehicle detection and tracking using gaussian mixture model and kalman filter. Proceedings of the 2016 International Conference on Computational Intelligence and Cybernetics.

[23] Wojke N., Bewley A., Paulus D. Simple online and realtime tracking with a deep association metric. Proceedings of the 2017 IEEE International Conference on Image Processing (ICIP).

[24] Hou X., Wang Y., Chau L.P. Vehicle tracking using deep SORT with low confidence track filtering. Proceedings of the 2019 16th IEEE International Conference on Advanced Video and Signal Based Surveillance (AVSS).

[25] Ammar A., Koubaa A., Ahmed M., Saad A., Benjdira B. (2021). Vehicle detection from aerial images using deep learning: A comparative study. Electronics.

[26] Ham S.W., Park H.C., Kim E.J., Kho S.Y., Kim D.K. (2020). Investigating the Influential Factors for Practical Application of Multi-Class Vehicle Detection for Images from Unmanned Aerial Vehicle using Deep Learning Models. Transp. Res. Rec..

[27] Wu Q., Zhou Y. Real-Time Object Detection Based on Unmanned Aerial Vehicle. Proceedings of the 2019 IEEE 8th Data Driven Control and Learning Systems Conference (DDCLS).

[28] Liu X., Yang T., Li J. (2018). Real-time ground vehicle detection in aerial infrared imagery based on convolutional neural network. Electronics.

[29] Xu Y., Yu G., Wang Y., Wu X., Ma Y. (2017). Car detection from low-altitude UAV imagery with the faster R-CNN. J. Adv. Transp..

[30] Xia G.S., Bai X., Ding J., Zhu Z., Belongie S., Luo J., Datcu M., Pelillo M., Zhang L. DOTA: A large-scale dataset for object detection in aerial images. Proceedings of the IEEE Conference on Computer Vision and Pattern Recognition.

[31] Turner S.M., Eisele W.L., Benz R.J., Holdener D.J. (1998). Travel Time Data Collection Handbook.

[32] Wang Z. (2004). Using floating cars to measure travel time delay: How accurate is the method?. Transp. Res. Rec..

[33] Cathey F.W., Dailey D.J. (2003). Estimating corridor travel time by using transit vehicles as probes. Transp. Res. Rec..

[34] Bertini R.L., Tantiyanugulchai S. (2004). Transit buses as traffic probes: Use of geolocation data for empirical evaluation. Transp. Res. Rec..

[35] Zou L., Xu J.M., Zhu L.X. Arterial speed studies with taxi equipped with global positioning receivers as probe vehicle. Proceedings of the 2005 International Conference on Wireless Communications, Networking and Mobile Computing.

[36] Coifman B., Kim S. (2009). Measuring freeway traffic conditions with transit vehicles. Transp. Res. Rec..

[37] Thornton D., Coifman B. (2015). Signal progression impacts on transit buses as travel time probes. J. Transp. Eng..

[38] Haseman R.J., Wasson J.S., Bullock D.M. (2010). Real-time measurement of travel time delay in work zones and evaluation metrics using bluetooth probe tracking. Transp. Res. Rec..

[39] Martchouk M., Mannering F., Bullock D. (2011). Analysis of freeway travel time variability using Bluetooth detection. J. Transp. Eng..

[40] Tufuor E.O., Rilett L.R. (2019). Validation of the Highway Capacity Manual urban street travel time reliability methodology using empirical data. Transp. Res. Rec..

[41] Wardrop J.G., Charlesworth G. (1954). A Method of Estimating Speed and Flow of Traffic from a Moving Vehicle. Proc. Inst. Civ. Eng..

[42] Sohn K., Kim D. (2008). Dynamic origin–destination flow estimation using cellular communication system. IEEE Trans. Veh. Technol..

[43] Caceres N., Romero L.M., Benitez F.G., del Castillo J.M. (2012). Traffic flow estimation models using cellular phone data. IEEE Trans. Intell. Transp. Syst..

[44] Ma W., Qian S. (2021). High-resolution traffic sensing with probe autonomous vehicles: A data-driven approach. Sensors.

[45] Kuhn H.W. (1955). The Hungarian method for the assignment problem. Nav. Res. Logist. Q..

[46] Kalman R.E. (1960). A new approach to linear filtering and prediction problems. ASME J. Basic Eng..

[47] Szeliski R. (2010). Computer Vision: Algorithms and Applications.

